# Muscle strength and body composition in severe obesity

**DOI:** 10.6061/clinics/2017(05)03

**Published:** 2017-05

**Authors:** Alexandre Vieira Gadducci, Roberto de Cleva, Gabriela Correia de Faria Santarém, Paulo Roberto Santos Silva, Julia Maria D’Andréa Greve, Marco Aurélio Santo

**Affiliations:** I Departamento de Gastroenterologia, Hospital das Clinicas HCFMUSP, Faculdade de Medicina, Universidade de Sao Paulo, Sao Paulo, SP, BR; II Instituto de Ortopedia e Traumatologia, Hospital das Clinicas HCFMUSP, Faculdade de Medicina, Universidade de Sao Paulo, Sao Paulo, SP, BR

**Keywords:** Obesity, Body Composition, Muscle Strength

## Abstract

**OBJECTIVE:**

The aim of our study was to evaluate associations between maximum voluntary contraction torques of the lower limbs and body composition for subjects with severe obesity.

**METHODS:**

Body composition was evaluated by bioelectrical impedance analysis, and maximum voluntary contraction torques of the lower limbs were measured using an isokinetic dynamometer. One hundred thirty-two patients were enrolled (100 females and 32 males). Eighty-seven patients had a body mass index between 40 and 49.9 kg/m^2^ (the A group), and 45 patients had a body mass index between 50 and 59.9 kg/m^2^ (the B group).

**RESULTS:**

Absolute extension and flexion torques had weak associations with fat-free mass but a moderate association with absolute extension torque and fat-free mass of the lower limbs. There were no significant differences between the A and B groups with respect to absolute extension and flexion torques. For the A group, absolute extension and flexion torques were moderately associated with fat-free mass and with fat-free mass of the lower limbs. For the B group, there were only moderate associations between absolute extension and flexion torques with fat-free mass of the lower limbs.

**CONCLUSIONS:**

Our findings demonstrate that both groups exhibited similar absolute torque values. There were weak to moderate associations between absolute extension and flexion torques and fat-free mass but a moderate association with fat-free mass of the lower limbs. Individuals with severe obesity should strive for greater absolute torques, fat-free mass and especially fat-free mass of the lower limbs to prevent functional limitations and physical incapacity.

## INTRODUCTION

Severe obesity 1 is associated with arterial hypertension, dyslipidemia, diabetes 2, metabolic syndrome 3, musculoskeletal problems such as osteoarthritis 4, reduced mobility 5 and gait disturbances 6. Excessive fat mass (FM) contributes to changes in the strength and endurance of skeletal muscles 7. Mid-thigh muscle mass is approximately 2.5 times that of fat mass, but individuals who are obese have increased intra- (fat within muscle cells) and intermuscular fat (fat between muscle cells) 8, establishing a negative influence on force generation capacity and functional independence. The functional capacity of a skeletal muscle can be assessed based on the muscle’s ability to produce force. Absolute strength 9 (the force exerted by an individual) is important for the execution of normal daily activities. Relative strength 9 (an individual’s force relative to body mass) is particularly useful for comparing individuals with different body dimensions.

Certain studies of obese patients have demonstrated that these subjects exhibit elevated absolute maximum voluntary contraction (MVC) torques 10 but reduced muscle strength relative to body weight 11 and to fat-free mass (FFM) than normal weight patients 12.

Nevertheless, no studies have evaluated the associations between lower limb MVC torques and body composition in patients with severe obesity. We hypothesized that segmental body composition might better determine the association between FFM of the lower limbs and MVC torque.

The aim of our study was to evaluate the associations between MVC torques of the lower limbs and body composition for subjects with severe obesity.

## METHODS

We consecutively evaluated 155 patients with severe obesity undergoing bariatric surgery in the Bariatric and Metabolic Surgical Unit of the Hospital das Clínicas, University of São Paulo Medical School. The inclusion criteria were age between 18 and 60 years, a body mass index (BMI) between 40 and 60 kg/m^2^ and a Timed Up and Go (TUG) ≤10 seconds. The exclusion criteria were as follows: patients with functional disability (TUG >10) 13, treatment with steroid medication for any reason or the use of artificial devices such as an orthosis or a prosthesis. Twenty-three patients were excluded: 5 subjects with BMI <40 kg/m^2^, 7 with BMI >60 kg/m^2^, and 11 with musculoskeletal disorders (7 subjects with TUG results >10 seconds and 4 subjects with artificial devices). Then, 132 patients (100 females and 32 males) were enrolled in the study, including 87 patients with a BMI between 40 and 49.9 kg/m^2^ (the A group) and 45 patients with a BMI ≥50 kg/m^2^ (the B group).

Body composition was determined by bioelectrical impedance analysis (BIA) under constant conditions (with subjects appropriately hydrated and at the same time of day). The body composition analyzer (InBody230, Biospace Co., Gangnam-gu, Seoul, South Korea) was a segmental impedance device that uses a tetrapolar 8-point tactile electrode system, and the measured weight range was 10 to 250 kg. Impedance measurements were performed by utilizing 2 different frequencies (20 and 100 kHz) at each segment (the right arm, left arm, trunk, right leg, and left leg). The participant was positioned in an orthostatic position on a platform with lower electrodes for the feet and two brackets (the upper electrodes) gripped on hands. Data output was calculated in percentages (%) and included FM, FFM, trunk FFM, and appendicular FFM (the sum of the FFM values for the right arm, left arm, right leg, and left leg). The Biodex^®^ Multi-joint System 3 dynamometer (Biodex Medical Systems, Inc., Shirley, NY, USA) was used to measure isokinetic extension (Ext) and flexion (Flex) MVC torques for both legs.

The dynamometer was calibrated before each test, and a strap was used to attach the dynamometer’s arm 3 cm above the lateral malleolus. Straps were also applied across the chest, pelvis and mid-thigh regions. Participants remained seated on the dynamometer chair, with the hip and knee joints at 90° flexion, and performed four submaximal contractions involving Ext and Flex of the knees during a warm-up period to familiarize themselves with their MVCs and produce consistent results. Participants then executed two series of four uninterrupted repetitions of Ext and Flex of both legs, first with the dominant member and subsequently with the non-dominant member, at an angular velocity of 60°/s, with a 60-second interval between series. During the testing period, standardized encouragement (e.g., “You are doing well”) was provided to all volunteers to ensure that strength during the contractions was maximized 14. The MVC variables that were assessed included absolute Ext and Flex torques (Nm), Ext and Flex torques relative to the body weight (Nm/Bw) and Ext and Flex torques relative to FFM (Nm/FFM) 15,16.

### Statistical analyses

The sample size was estimated based on an expected effect size of 10% for the relationships between FFM and the Ext and Flex MVC torques and a significance threshold of 5% (*p*<0.05). The calculated minimum sample size was 132 subjects. All data are presented as the mean ± standard deviation, median, first quartile and third quartile. Unpaired t-tests were used for comparisons between groups when normality was not rejected by the Anderson Darling test. In case of rejection of normality, we used the Mann-Whitney test when the variables were homogeneous and the t test and Brunner Munzel test when the variables were heterogeneous. Homogeneity (or homoscedasticity) was verified by the Bartlett test. Associations were evaluated using Pearson and Spearman correlations.

### Ethical considerations

Informed consent was obtained from all participants included in this study. All study procedures were conducted in accordance with the ethical standards of relevant institutional and/or national research committees and thus satisfied the standards set forth in the Declaration of Helsinki in its revised version from 1975 and its amendments in 1983, 1989, and 1996. This study was approved by the Hospital das Clínicas Ethical Committee, University of São Paulo Medical School (no. 01038912.6.0000.0068).

## RESULTS

The anthropometric characteristics, body composition and absolute and relative MVC torques of individuals are listed in Table 1. There were no differences between the dominant and non-dominant lower limbs with respect to absolute extension (156.76±43.67; 156.15±46.2, *p*=0.992) or flexion (72.52±23.56; 71.45±21.6, *p*=0.901). Therefore, only the dominant member was considered for analysis.

The anthropometric characteristics, body composition and absolute and relative MVC torques of subjects grouped by obesity grade are provided in Table 2. There were no significant differences between the two groups with respect to age or height. Significant between-group differences were detected for all body composition variables. Both groups exhibited similar mean values of absolute Ext and Flex MVC torques. However, Ext MVC torque relative to body weight (BW) and FFM was significantly reduced in the B group.

The correlations between absolute MVC torques and total and segmental body composition for the patient groups are listed in Table 3. The absolute Ext and Flex MVC torques had weak associations with FFM. However, there was a moderate association with absolute extension torque and fat with free mass of lower limbs (FFMLL). For the A group, the absolute Ext and Flex MVC torques had moderate associations with FFM and FFMLL. For the B group, the absolute Ext and Flex MVC torques had a weak association with FFM and a moderate association with FFMLL.

## DISCUSSION

Obesity is associated with musculoskeletal disorders, such as mechanical overload 17, low muscular conditioning 18 and the increased prevalence of musculoskeletal pain 19_._ The lower limb muscles (flexors and extensors) are responsible for balance control 20, mobility and the ability to execute activities of daily life 21.

Excessive FM is regarded as the main determinant of reduced relative muscle strength 22, pain in joints of the lower limbs and functional limitations, especially in individuals with severe obesity 17. Nevertheless, there are no studies correlating the FFM and FFM of the lower limbs with MVC torque 21. Previous studies have evaluated MVC torques of the lower limbs in obese patients 9,10,12 at low angular velocities (60°/s) due to the recruitment of a relatively large number of motor units 23. The results represent the evaluated muscles’ maximum capacity to produce force. Furthermore, patients with severe obesity have preponderance of muscle fibers (type IIb) related to short-duration, high-intensity and low-repetition activities that can be more precisely evaluated at low angular velocities 24,25.

Absolute MVC torques might be greater for obese individuals than for normal-weight individuals 10,12. The lower limb muscles are responsible for cushioning, stabilizing the knee joint 26, and providing high levels of concentric strength during repetitive movements; these muscles support three times the body weight during walking and six times the body weight when climbing stairs 27.

A progressive increase in body mass would require a progressive increase in absolute lower limb Ext and Flex strength. However, in this study, the B group did not exhibit greater absolute Ext and Flex MVC torques than the A group. These results suggest that the B group had limited absolute MVC torques (that is, that these torques had plateaued). The reduced relative Ext MVC torque observed in the B group in comparison with that of the A group could be explained by functional limitations 28 associated with a lack of physical activity due to excessive body mass, disuse and muscular atrophy 29; a consequence of this lack of activity is compromised independence in activities of daily living.

The correction of MVC torque for BW and FFM allows for comparisons among individuals with different body masses 15,16 and improved representations of the support loads that lean mass must bear in obese subjects.

The B group presented with more severe losses in FFM than the A group; this change in body composition was associated with lower relative MVC torques and contributes to a reduced level of physical activity 28.

In previous studies, weak associations (r=0.29 - 0.49) between lower limb strength and FFM were observed in obese women 8,10,12. However, in our series, we observed weak to moderate associations between MVC torques and FFM (r=0.47 - 0.6) and moderate associations with FFMLL (r=0.53 - 0.67). Our results suggest that fat-free mass (mainly in the lower limbs) is crucial for increasing the absolute MVC torques. The reduced FFMLL is a key determinant of functional limitations, physical inactivity and the emergence of comorbidities in patients with severe obesity 30. Physical exercises specifically planned for such patients may prevent losses of muscle strength 31.

## AUTHOR CONTRIBUTIONS

Gadducci AV was responsible for the preparation of the study, data collection, research execution and manuscript drafting. de Cleva R was responsible for the preparation of the study, research execution and manuscript drafting. Santarem GC was resp nsible for the data collection and manuscript drafting. Silva PR was responsible for the preparation of the study, research execution and manuscript drafting. Greve JM was responsible for the manuscript drafting. Santo MA was responsible for the resea ch execution and manuscript drafting.

## Figures and Tables

**Table 1:** Anthropometric characteristics, body composition, and absolute and relative maximum voluntary contraction torques for obese patients.

	Mean ± SD	Median	1° Q - 3° Q
Age (years)	40.5±9.79	41	33.25-47.75
Height (cm)	1.62±0.1	1.61	1.56-1.67
Weight (kg)	126.13±19.64	122.35	111.93-136.85
IMC	47.65±4.89	47.2	43.95-51.18
FFM%	49.27±4.18	48.27	46.5-51.47
FFMLL%	50.72±5.46	49.93	46.96-53.99
FM%	50.73±4.18	51.73	48.53-53.5
FMLL%	49.28±5.46	50.07	46.01-53.04
Ext (Nm)	156.45±43.57	150.75	123.88-183.48
Ext (Nm/Bw)	123.53±29.4	122.9	102.06-141.15
Ext (Nm/FFM)	250.91±47.67	249.14	219.49-278.68
Flex (Nm)	71.98±21.98	67.75	55.84-82.2
Flex (Nm/Bw)	56.95±15. 68	56.62	46.15-67.35
Flex (Nm/FFM)	115.58±26.87	114.89	97.52-132.42

Note: SD: standard deviation; BMI: body mass index; FFM: fat-free mass; FM: fat mass; Ext: extension; Flex: flexion; Nm: newton-meter; Nm/Bw: newton-meter/body weight; Nm/FFM: newton-meter/fat-free mass.

**Table 2:** Anthropometric characteristics, body composition, and absolute and relative maximum voluntary contraction torques for patient groups determined based on the obesity grade.

	A Group (n = 87)	Median	1° Q - 3° Q	B Group (n = 45)	Median	1° Q - 3° Q	*p*
Age (years)	41.8±9.2	42	35-48	38 ± 10.4	37	31.2-46.2	0.046^T^
Height (cm)	163±0.1	162	156-168	162±0.1	159	155-166	0.343^M^
Weight (kg)	119±15.8	115.5	107.1-126.2	140±18.6	136.5	127.8-150.8	<0.001^M^
BMI (kg/m^2^)	44.7±2.8	44.8	42.4-47.2	53.3±2.3	53	51.2-55	<0.001^M^
FFM%	50.6±4.3	49.5	47.4-53.1	46.6±2.3	46.5	44.9-47.6	<0.001^M^
FFMLL%	52.53±4.9	51.7	48.9-55.5	47.19±4.6	46.4	44.7-49.5	<0.001^M^
FM%	49.4±4.3	50.4	46.9-52.5	53.4±2.3	53.5	52.3-55	<0.001^M^
FMLL%	47.47±4,9	48.2	44.4-51.1	52.81±4.6	53.5	50.4-55.2	<0.001^M^
Ext. (Nm)	156.4±45	151.5	123.8-175.2	156.4±41	148.2	125.2-189.9	0.971^M^
Ext (Nm/Bw)	130±30.7	131.9	112-145.2	110.9±22.2	108.2	96.2-130.8	<0.001^T^
Ext. (Nm/FFM)	257.8±49	259.5	223.8-285.4	237.4±42	235.4	201.5-268.5	0.015^T^
Flex (Nm)	71.5±22	67.1	56.1-83.1	72.8±22	70	55-82	0.79^M^
Flex (Nm/Bw)	59.6±16.1	59.9	48.8-68.7	51.7±13.5	50.4	43.5-61.1	0.004^T^
Flex (Nm/FFM)	118.1±26.8	116	99.3-134.8	110.6±26.6	106.9	93.2-131.7	0.135^T^

Note: BMI: body mass index; FFM: fat-free mass; FFMLL: fat-free mass of the lower limbs; FM: fat mass; FMLL: fat mass lower limbs; Ext: extension; Flex: flexion; Nm: newton-meter; Nm/Bw: newton-meter/body weight; Nm/FFM: newton-meter/fat-free mass;

T: Test t;

M: Mann-Whitney.

**Table 3:** Correlation between muscle strength and total and segmental body composition for patient groups determined based on the obesity grade.

FFM	All patients	*p*	A Group	*p*	B Group	*p*
	r (95% CI)		r (95% CI)		r (95% CI)	
Ext (Nm)	0.48 (0.35; 0.63)	<0.001^S^	0.6 (0.49; 0.78)	<0.001^S^	0.47 (0.16; 0.7)	0.001^P^
Flex (Nm)	0.46 (0.33; 0.6)	<0.001^S^	0.57 (0.42; 0.76)	<0.001^S^	0.48 (0.26; 0.79)	0.001^S^
**FFMLL**						
Ext (Nm)	0.57 (0.45; 0.71)	<0.001^S^	0.67(0.56; 0.85)	<0.001^S^	0.64 (0.39; 0.8)	<0.001^P^
Flex (Nm)	0.47 (0.33; 0.62)	<0.001^S^	0.56 (0.4; 0.77)	<0.001^S^	0.53 (0.33; 0.82)	<0.001^S^

Note: FFM: fat-free mass; FFMLL: fat-free mass of the lower limb; Nm: newton meter; Ext: extension; Flex: flexion;

S: correlation spearman;

P: Pearson correlation.
